# Emerging Micro Manufacturing Technologies and Applications, 3rd Edition

**DOI:** 10.3390/mi17091001

**Published:** 2026-08-25

**Authors:** Nikolaos Tapoglou

**Affiliations:** Laboratory of Machine Elements and Machine Design, School of Mechanical Engineering, Aristotle University of Thessaloniki, 54124 Thessaloniki, Greece; ntapoglou@meng.auth.gr

The field of micro-manufacturing continues to advance at a rapid pace, driven by the increased demand for components and assemblies with smaller size and ever tightening tolerances. The economic impact of the sector is hard to estimate as it transpires across many industries and applications. Traditionally, micro-components have been associated with the electronics, watchmaking and semiconductor industries, where miniaturization has long been the principal driver. In recent years, however, the industry has expanded into aerospace, biomedical devices, and precision instrumentation, and with it the range of materials and processes now employed.

As features cross into the micrometer range, mechanisms that govern traditional machining processes no longer apply and phenomena once considered secondary come to dominate process behavior. The size effect is the primary cause behind this behavior, especially in machining processes. A series of processes have been adapted and developed specifically to tackle issues with manufacturing components in the microscale range [1,2]. On the process performance level, tool wear, burr formation, and residual stress evolution take on heightened significance at these scales, demanding renewed and increasingly interdisciplinary investigation—spanning experimental, numerical, and data-driven methods [3,4].

Following the success of the first two editions of this Special Issue [5,6], this third edition of the Special Issue “Emerging Micro-Manufacturing Technologies and Applications” brings together nine contributions that span experimental, numerical, and data-driven approaches across a range of processes, including precision and micro-milling, micro-electrical-discharge and electrochemical jet machining, abrasive-film microfinishing, stereolithography-based additive manufacturing, and molecular-scale polishing simulation.

Kacalak et al. [Contribution 1] investigated the micro-finishing of surfaces with the use of abrasive films. The research introduced three concepts of micro-finishing apparatus focusing on external cylindrical surfaces. The first concept presented used a dual arm design to reduce workpiece deformation and improve the throughput of the process. The second design introduced a submerged processing route where a processing fluid bath is used to fully submerge the component while processing, thus improving heat dissipation and accuracy. The third design introduced a processing design with graphite-impregnated films, simultaneously smoothing the surface and forming a wear-resistant carbon coating. The micro-finishing methods were validated on the hard-to-machine Nimonic 80A superalloy.

Xue et al. [Contribution 2] presented a novel molecular dynamics-based simulation model for the investigation of nano-polishing of nickel–phosphorus (NiP) alloys. Using a silica abrasive, they focused on the influence of phosphorus content, polishing depth, and speed on material removal mechanisms, surface morphology, residual stress distribution, and temperature variation. The results indicated that increased polishing depths and speeds reduce surface quality and increase residual stress and temperature. Phosphorus content was the dominant factor influencing surface morphology.

Zhang et al. [Contribution 3] studied the optimization of micro-EDM processes for the generation of microgrooves. The research focused on actively compensating the electrode wear during the process. In order to achieve this, an orthogonal design of experiments and gray relational analysis were applied to simultaneously optimize machining time and electrode wear. Based on the experimental results, the method demonstrated a reduction in machining time by 10% and a reduction in axial and radial wear of the electrode by 2 and 11% respectively.

Huang et al. [Contribution 4] presented a comprehensive review of electrochemical jet machining (ECJM). The process has been gaining traction due to the ability to process complex, fine feature geometries on hard-to-machine materials. The review outlines the fundamental principles and process parameters of ECJM, the influence of electrolyte type on machining accuracy and surface quality, and strategies for mitigating stray corrosion. The review covers a series of hybrid and energy-assisted variants—ultrasonic-, laser-, magnetism-, and abrasive-assisted ECJM. These include ultrasonic-assisted electrochemical jet machining (UECJM), laser-assisted electrochemical jet machining (LAECJM), magnetic field-assisted electrochemical jet machining (MECJM) and abrasive-assisted electrochemical jet machining (AECJM). The application of these methods has shown the potential to enhance material removal rate, precision, and surface finish.

Zhou et al. [Contribution 5] investigated the impact of scratch-induced microscale surface roughness on the signal transmission in RF coaxial connectors. The study focused on the elastic pin–receptacle contact interface of precision 2.92 mm connectors. The controlled experimental procedure they performed identified two competing degradation mechanisms: overly smooth surfaces form insufficient micro-contacts and raise contact resistance, while excessively rough surfaces concentrate current in narrow constriction paths, with signal integrity declining as roughness increases across the moderate range and these effects amplified in multi-connector cascades.

Metelli et al. [Contribution 6] presented a Finite Element Method (FEM)-based design and manufacturing approach of a ratchet click mechanism for mechanical watch movements. Based on observations from fractured original parts, the authors developed an elastoplastic contact simulation FEM model to inform the redesign of the components, leading to an optimized configuration with reduced peak stresses, particularly on the wheel tooth, keeping the ratchet within its elastic range. The components were micromachined with HSM practices. A validation campaign of forty watches was run for circa 400 h each with no failures observed.

AL-Khafaji et al. [Contribution 7] presented a hybrid intelligent framework for modeling and optimization of the stereolithography (SLA) 3D printing process for an ABS photopolymer. The work proposed a novel algorithm based on a hybridized custom MLFE with a PSO framework to create a predictive model of the process. The models, tuned with experimental data, predicted five performance responses, namely ultimate tensile strength, yield strength, Young’s modulus, Shore D hardness, and surface roughness. The work demonstrates that the hybrid fuzzy–PSO approach offers an accurate and interpretable alternative to black-box models for the modeling and multi-objective optimization of SLA printing.

Korpysa [Contribution 8] investigated precision milling of AZ91D magnesium alloy through an experimentally based campaign. The experiments performed were observed through a high-speed camera recording at 10,000 FPS to observe the cutting performance in a range of feeds. Based on the recordings it was observed that the two flutes of the end mill did not perform uniformly, with one flute producing larger chips and the other generating fine chips or fragments. The study also indicated that large burrs formed at low feeds per tooth. A feed of 2.7 µm/tooth was identified as a transition limit between plowing-dominant and shearing-dominant regimes.

Zhu et al. [Contribution 9] conducted an experimental investigation into the milling of high-aspect-ratio holes in titanium alloy Ti-6Al-4V. In the research an orthogonal DOE was employed to examine the effects of milling depth, feed per tooth, and cutting speed on cutting force and burr formation. Milling depth was found to be the governing factor in milling force. Optimal processing parameters derived by the experimental investigation were a milling depth of 60 μm, feed per tooth of 2 μm/z, and cutting speed of 31 m/min. These parameters offered a reduced milling force of 3.61 N, a 91.74% decrease relative to the maximum-force condition, while substantially lowering entrance burr width and improving geometrical accuracy.

The contributions of this Special Issue highlighted a series of emerging trends that are gaining traction in the micro-manufacturing domain [7,8]. The first stream concerns the predictive frameworks developed by researchers to model and predict the behavior of systems; the extension towards building trustworthy digital twins and adaptive control is a natural successor. The second stream is the development of data efficient machine learning models able to provide accurate predictions using small datasets. The third stream is the integration of hybrid and additive processes to improve throughput. 
